# “Princess and the pea” – an assessment tool for palpation skills in postgraduate education

**DOI:** 10.1186/s12909-019-1619-6

**Published:** 2019-05-30

**Authors:** Rainer Kamp, Andreas Möltner, Sigrid Harendza

**Affiliations:** 1 Academy of Medical Education of the Medical Council Westphalia-Lippe, Ärztekammer Westfalen-Lippe and Kassenärztliche Vereinigung Westfalen-Lippe, Münster, Germany; 2 Ruprecht-Karls-University, Center of Excellence for Assessment in Medicine – Baden Württemberg, Heidelberg, Germany; 3 III. Department of Internal Medicine, University Medical Center Hamburg-Eppendorf, Hamburg, Germany; 4 Universitätsklinikum Hamburg-Eppendorf, III. Medizinische Klinik Martinistr. 52, 20246 Hamburg, Germany

**Keywords:** Assessment, Palpation skill, Physical examination, Postgraduate medical education, Osteopathic medicine, Manual medicine

## Abstract

**Background:**

In osteopathic medicine, palpation is considered to be the key skill to be acquired during training. Whether palpation skills are adequately acquired during undergraduate or postgraduate training is difficult to assess. The aim of our study was to test a palpation assessment tool developed for undergraduate medical education in a postgraduate medical education (PME) setting.

**Methods:**

We modified and standardized an assessment tool, where a coin has to be palpated under different layers of copy paper. For every layer depth we randomized the hiding positions with a random generator. The task was to palpate the coin or to determine that no coin was hidden in the stack. We recruited three groups of participants: 22 physicians with no training in osteopathic medicine, 25 participants in a PME course of osteopathic techniques before and after a palpation training program, 31 physicians from an osteopathic expert group with at least 700 h of osteopathic skills training. These experts ran the test twice to check for test-retest-reliability. Inferential statistical analyzes were performed using generalized linear mixed models with the dichotomous variable “coin detected / not detected” as the dependent variable.

**Results:**

We measured a test-retest reliability of the assessment tool as a whole with 56 stations in the expert group of 0.67 (*p* <  0.001). For different paper layers, we found good retest reliabilities up to 300 sheets. The control group detected a coin significantly better in a depth of 150 sheets (*p* = 0.01) than the pre-training group. The osteopathic training group showed significantly more correct coin localizations after the training in layer depths of 200 (*p* = 0.03) and 300 sheets (*p* = 0.05). This group also had significantly better palpation results than the expert group in the depth of 300 sheets (*p* = 0.001). When there was no coin hidden, the expert group showed significantly better results than the post-training group (*p* = 0.01).

**Conclusions:**

Our tool can be used with reliable results to test palpation course achievements with 200 and 300 sheets of paper. Further refinements of this tool will be needed to use it in complex assessment designs for the evaluation of more sophisticated palpatory skills in postgraduate medical settings.

## Background

Physical examination (PE) has been one of the cornerstones of the evaluation and treatment of patients for thousands of years [1, 2]. Although training of PE is a core element in undergraduate medical education (UME) it is only irregularly taught in postgraduate medical education (PME) [3]. PE training-aspects are also underemphasized when PE is formally assessed [4]. Palpation is one of four core skills in PE, the others being inspection, percussion, and auscultation. In osteopathic medicine, palpation is considered to be the key skill to be acquired during training. According to the glossary of osteopathic terminology “palpation is the application of the fingers to the surface of the skin or other tissues, using varying amounts of pressure, to selectively determine the condition of the parts beneath” [5]. Palpation is a complex skill, which is partly reproducible but depends on individual factors. Furthermore, between the ages of 20 and 80, the somatosensory ability decreases physiologically by an average of approximately 1% per year, but this natural decrease is evidently slowed in practicing physiotherapists and osteopaths by the demands placed on the haptic system [6, 7].

Touch is extremely difficult to convey verbally [8]. It has been shown that osteopathic palpation shows a fair intra-observer reliability and a low inter-observer reliability even for palpation of anatomic landmarks at the surface of the body [9]. When assessing the reliability of a test, inter-examiner agreement is of greater importance than intra-examiner agreement [10]. There are many factors that may lead to inter-examiner inconsistencies, such as the examiner’s expectations and clinical diagnostic skills, examiner fatigue, the degree of asymmetry, movement of the subject, etc. [11]. There is a relationship between search technique, force, and accuracy, as Laufer showed for clinical breast examination (CBE). Her group was able to define a specific range of palpation forces that increase the likelihood of an accurate CBE [12]. Objective assessment of palpation requires assessment methods that are valid, reliable, fair, defensible and evidence-based [13].

In medical education, training instruments for palpation range from expensive and carefully engineered technical apparatus [14] to simple, self-developed creative and cheap tools [15]. To achieve layer by layer palpation skills through several types of tissues including skin, fascia, muscle, and bone, it is necessary to learn to apply different levels of pressure with fingers and hands. Loh and Amsler assessed the role of training and practice in an osteopathic medical student population by locating a dime placed under sheets of copy paper [16]. Both, paper depth and experience (i.e. beginning vs. end of the term) were statistically significant determinants of the number of correct responses. Hence, as a cheap and easy to produce testing tool it might be a useful assessment instrument for palpation training in undergraduate and postgraduate medical education.

However, sheets of copy paper do not represent the texture of living tissues. But any simulated assessment method can never be as perfect as reality [17]. In reality, assessment always involves a compromise [18]. Quantitative and qualitative information from single assessments is first and foremost used to promote learning. But it is also aggregated across a large sample of contexts and assessors in order to obtain an overall picture of a trainee’s progress [19]. A higher degree of objectivity in a practical assessment makes the process and the results more transparent. The aim of our study was to test the palpation assessment tool developed by Loh and Amsler [16] in a PME setting to answer the following research questions: Are the results reproducible? Can the instrument be used for standard setting with fully trained experts in osteopathic medicine?

## Methods

### Instrument

Following the idea of Loh and Amsler we hid a coin under a stack of copy paper. For standardization, we modified the original concept in the following way: we used a European cent coin (mass 2.30 g, thickness 1.67 mm, diameter of 16.25 mm, edges smooth, material coppered steel [20]), and a stack of copy paper (Plano-Speed, DIN A 4, 80 g/qm) with 500 sheets. We hid the coin under layers of either 50, 100, 150, 200, 300, 400 or 500 sheets. The task is to locate the coin or to determine that no coin is hidden in the stack. If a coin is located, it has to be marked on the top (cover) sheet of the stack with a red marking dot (Avery, diameter 12 mm). The cover sheet bears the station number and a participant’s personal code (anonymized). A frame of 1 cm is drawn from each side of the sheet to avoid false palpation of natural bumps and bulges. We avoided any further visual orientation on the cover sheet.

For reasons of dot equivalence, we designed a hexagon (Microsoft Office 365, version 2016) in the center of a sheet of copy paper (carrier sheet) to determine the coin’s position. We defined seven possibilities to fix the coin with simple glue: the six corners of the hexagon and the geometrical middle-point of the hexagon. These positions are numbered from 1 to 7, position 1 being the 12 o’clock position and then clockwise to position 6, position 7 being in the middle of the hexagon. For every layer depth we randomized the hiding positions with a random generator [21]. For every layer, we prepared five carrier sheets with a coin and three with no coin following the random column. Then we transferred the paper stack into a conventional DIN A4 cardboard box (measures: 305 × 215 × 100/100 mm) to prevent larger displacement of paper sheets. This allows to place the examining hands only on top of the paper stack preventing participants from putting their hands on both sides of the stack and getting information from bimanual compression. Following the results of a random generator again (seven layers, eight possibilities, 56 stations), the stacks are placed in a round course. This step is blinded to the facilitator and the time-keepers.

### Study design

Between February 2017 and January 2018, we recruited participants from 3 groups: 1) 22 physicians (nine females,13 males, age: 27 to 55 years), with no training in osteopathic palpatory skills or manual medicine from a medical education master course at the medical faculty of Heidelberg university. The minimum postgraduate medical training was 3 years (control group). 2) 25 participants (4 females, 21 males, age 40 to 63 years) in a postgraduate medical education (PME) course in osteopathic techniques were invited to take the test. This group was tested before starting their palpation training program and after having finished it. All were board certified in a medical specialty and had had a postgraduate training in Manual Medicine of 340 h before. In the post training assessment, we had four dropouts, two participants were ill, one was on duty service and one could not participate for unknown reasons, leaving 21 participants (four females, 17 males) who completed the pre- and post-training testing. 3) 31 volunteers (four females, 27 males, age 36 to 59 years) from an expert group with a standard of 700 h of osteopathic skills training (European Register of Osteopathic Physicians (EROP) Standard) were recruited during the annual teachers meeting of German Society for Osteopathic Medicine (DGOM). They ran the test twice to check for test-retest-reliability. In summary, the study type is a three-group post-test comparison with one treatment group and two control groups (physicians with no training in palpatory skills or manual medicine, expert group with osteopathic training). Within the treatment group the design is a one-group pre-post-test design.

For organizational reasons, we took the measurements in three different locations, a university lecture hall, a college of osteopathic medicine, and a school for physiotherapists. In the latter two it was necessary to change rooms during the testing. The participants followed a round course. At each station, they had 45 s to palpate and mark the suspected coin or to decide that there was no coin in the stack and hence no mark needed. For analysis, we measured the distance between the middle of the cent-position and the middle of the marking dot, i.e.: 1) coin in the stack, coin detected and localization correct (≤ 3 cm) = correct, coin detected and localization incorrect (> 3 cm) = false, coin not detected = false; 2) no coin in the stack and no marking dot = correct, coin detected (marking dot) = false.

### Statistical analyses

Inferential statistical analyzes were performed using generalized linear mixed models (GLMM) with the dichotomous variable “coin detected / not detected” as the dependent variable (see above). Participant was a random factor and group a fixed factor. Since the variable to be explained is dichotomous, a logit function was selected as link function.The analyses were carried out independently for the different layer depths. The significance level was *p* = 0.05. No adjustments of the significance levels were necessary.

## Results

First, we report on the reliability of the instrument. For a cut of 3 cm from the coin detected we measured a test-retest reliability of the assessment tool as a whole with 56 stations in the expert group of 0.67 (*p* <  0.001). For the different layers, we found good retest reliabilities up to 300 sheets (Table 1). In a depth of 400 and 500 sheets we could not find a significant correlation coefficient.

**Table 1:** Retest-Reliabilities (expert group) for different paper depths

Depth (number of paper sheets)	Retest-Reliability	*p*-value
0	0.64	< 0.001
50	0.58	< 0.001
100	0.26	0.079
150	0.52	< 0.001
200	0.41	0.005
300	0.45	0.002
400	0.12	0.416
500	0.05	0.762

**Fig. 1:**
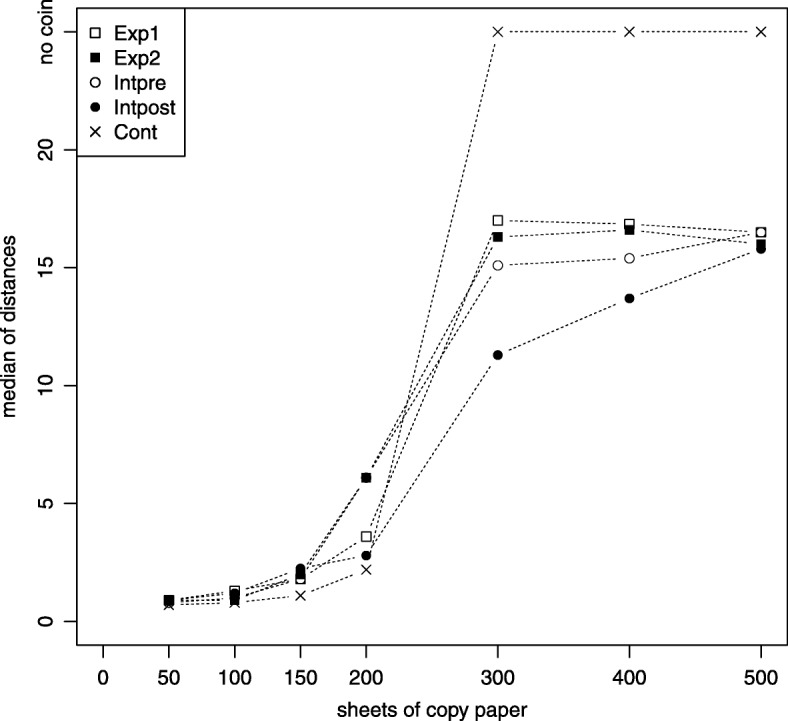
The median of the distances from the hidden coin to the marked positions in different layers of sheets of copy paper for the tested groups is shown. Groups: expert group’s first round (Exp1), expert group’s second round (Exp2), pre-training group (Intpre), post-training group (Intpost), control group (Cont)

The accuracy of coin detection was also measured (Fig. 1). The comparison of the results in the osteopathic training group before and after the training course showed significantly more correct localizations after the training in layer depths 200 (*p* = 0.03) and 300 sheets (*p* = 0.05). In the other depths, we could find a slight tendency of better results for the post-training group, which were not statistically significant. The post-training group showed significantly better palpation results than the expert group in the depth of 300 sheets (*p* = 0.001). When there was no coin hidden, the expert group showed significantly better results than the post-training group (*p* = 0.01). For the other layers we could not find any significant differences between these groups.

Furthermore, we compared the pre-training group with a control group. For detection of a coin in a depth of 150 sheets, the control group showed significantly better results (*p* = 0.01). For the correct decision that there was no coin under the paper, the control group showed significantly better results, too (*p* = < 0.001).

## Discussion

We hypothesized that a tool to palpate a coin under a stack of paper sheets, which had already been successfully used to test basic palpatory skills in an osteopathic medical student population [16], can be applied to assess the development of palpation skills in postgraduate medical education. Both, paper depth and experience (i.e. beginning vs. end of the term) had been statistically significant determinants of the number of correct responses by the students [16]. Our test as a whole with 56 stations showed good retest reliability in the expert group. This implies that the test as an assessment tool provides acceptable results.

However, the assessment of trainees and physicians who have higher levels of expertise, as in PME, presents particular challenges. Expertise is characterized by unique, elaborated, and well-organized bodies of knowledge that are often revealed only, when they are triggered by characteristic clinical patterns [22]. Apart from the more complex and clinically important learning experiences with the diagnostic palpation on other students or patients, students can also benefit from developing their visual, haptic, and spatial awareness skills in other learning environments [23]. Quantitative and qualitative information from single assessments is first and foremost used to promote learning [19]. Following Loh and Amsler [16] our test directs the learner’s focus on the idea of layer by layer palpation by applying different levels of pressure with fingers and hands.

When there was no coin hidden, the expert group showed better results than the post-training group. This might be the consequence of longer training and experience of the experts, but a mental bias of the post-training group is possible, too. In a sample of osteopaths and non-osteopaths, one third of the participants could detect a motion of 50 μm or less with their bare hands using a mechanical device with an actuator [24]. The only statistically significant difference between osteopaths and non-osteopaths in this study was the higher rate of false positive detections in reporting non-existing motions by the osteopaths. In this study, the authors argued that the osteopaths were trained to feel something and in case of doubt, they tended to report to feel something, which constitutes a mental bias. In our study, a similar mental bias might have caused the post-training group to claim the presence of a coin, when indeed there was no coin.

Diagnostic palpation is not only one of the hardest clinical skills to develop and teach but also to assess [23]. Development of performance metrics and assessments to ensure minimum performance standards is an important endeavour [8]. Therefore, good assessment requires a programmatic approach in a deliberate and arranged set of longitudinal assessment activities [25]. To augment the reproducibility between examiners, Lewit has proposed designing testing batteries with different tests [26]. The use of our tool in such a testing battery could contribute to the achievement of the goals mentioned above. In reality, assessment always involves a compromise [18]. Since the introduction of competency-based medical education (CBME), a common practice has been to reduce competencies to small units of behavior for the purposes of assessment. This “atomization” can lead to trivialization and may actually threaten validity [27]. It has to be considered that palpation is a complex skill, which is partly reproducible but depends on individual factors, e.g. perception and cognitive factors, the development of expertise and the clinical context [23]. Hence, recommendations for CBME include the use of multiple methods, multiple assessors, and assessment of palpation should be embedded within an effective educational system [28]. The focus is shifting towards formative assessment for learning, which provides a benchmark to orient the learner who is approaching a relatively unstructured body of knowledge [22]. Our testing tool might also have its place in formative assessment, especially in a pre-post course use.

One weakness of our study is, that a stack of copy paper is not comparable with human tissues. Our assessment tool focusses didactically on layer palpation, but it certainly is not content specific for palpating living tissues. However, it provides a measure of palpation skills within a certain range of paper layers. When standardizing the testing tool, we defined a distance of 3 cm from the coin as the limit between a correct and a false result. This is a best guess. The consistent results in most layers for the expert and post-training groups indicate the possible usefulness of this tool in a complex assessment of palpation. For reasons of randomisation and blinding, we used paper stacks of 500 sheets of copy paper. When there was no coin hidden, we could not assess a defined layer depth as for a hidden coin. Only the decision between “no coin inside = correct” and “coin inside = false” were possible. We will have to change the design for measuring the layer dependent palpation capability of participants. We did not use video surveillance to get an impression, how the participants executed their palpation. Therefore, we have no reproducible clues of the level of agreement between the different groups with respect to palpation techniques. Specific techniques that might have been handed down by tradition and taught as part of the routine clinical training produce different levels of accuracy [12]. If there is agreement between palpation techniques, the accuracy of palpatory findings can be significantly improved [29]. One strength of this study is that we proved the feasibility of the testing tool under realistic conditions.

In our study, we could show in real life situations in PME (measurements in three different locations, a university lecture hall, a college of osteopathic medicine, and a school for physiotherapists) that the tool produced reliable results for paper depths between 150 and 300 sheets of copy paper. We could contribute to overcome several of the limitations of the first study from Loh and Amsler [16]. Their study lacked a control group. We used a design with a control group of medical doctors with no osteopathic or MM background, and an expert group with an extended osteopathic training of a European (EROP) standard. Our strategy for further research of palpation skills will include looking at differences and similarities of the used palpation techniques. This might be helpful in bridging the gap between current palpation teaching and effective, evidence-based performance, as Laufer has pointed out for CBE [30].

## Conclusion

In postgraduate medical education, a measurement tool for palpatory skills from undergraduate medical education can be used successfully. Greater standardization was achieved by specific modification of the tool and it can be used with reliable results to test palpation course achievements of participants. Further refinements of this tool will be needed to use it in complex assessment designs for the evaluation of more sophisticated palpatory skills in postgraduate medical settings.

## Data Availability

All data and material are available in the manuscript.

## Abbreviations

CBE: Clinical breast examination
CBME: Competency-based medical education
DGOM: German Society for Osteopathic Medicine
EROP: European Register of Osteopathic Physicians
GLMM: Generalized linear mixed models
MM: Manual Medicine
PE: Physical examination
PME: Postgraduate medical education
UME: Undergraduate medical education
